# Bavachin enhances paclitaxel sensitivity in ovarian cancer cells through modulation of mitochondrial function and ER stress

**DOI:** 10.1080/19768354.2025.2520852

**Published:** 2025-06-30

**Authors:** Sang-Jin Lee, Kee K. Kim, Jin-Young Lee

**Affiliations:** a Department of Biological Sciences, College of Natural Sciences, Keimyung University, Daegu, Republic of Korea; b Department of Biochemistry, College of Natural Sciences, Chungnam National University, Daejeon, Republic of Korea

**Keywords:** Bavachin, ovarian cancer, mitochondrial dysfunction, ER stress, paclitaxel resistance

## Abstract

Bavachin, a bioactive phytoestrogen from *Psoralea corylifolia*, has shown promising therapeutic potential in various cancers, but its effects on ovarian cancer remain unexplored. In this study, we investigate the anti-cancer mechanisms of bavachin in ES2 and OV90 ovarian cancer cells and its potential to enhance paclitaxel sensitivity. Bavachin significantly inhibited cell proliferation and spheroid formation while inducing caspase-dependent apoptosis through modulation of Bcl-2 family proteins. Mechanistically, bavachin disrupted mitochondrial function by inducing membrane depolarization and calcium dysregulation, leading to comprehensive impairment of cellular bioenergetics including both oxidative phosphorylation and glycolysis. Furthermore, bavachin activated the endoplasmic reticulum stress pathway as evidenced by upregulation of GRP78, ATF4, PERK, and CHOP, and notably inhibited phosphorylation of ERK1/2 and p38 MAPK signaling pathways essential for tumor cells survival. Combination treatment with bavachin enhanced the cytotoxic effects of paclitaxel, showing synergistic anti-cancer activity in both cell lines. These findings demonstrate that bavachin effectively suppresses ovarian cancer growth through multiple mechanisms and may serve as a promising therapeutic agent, particularly in combination with conventional chemotherapy.

## Introduction

Ovarian cancer remains one of the most lethal gynecological malignancies worldwide, with high mortality rates primarily due to late diagnosis and development of chemoresistance. Although platinum-based chemotherapy combined with paclitaxel is the standard treatment, drug resistance frequently develops, highlighting the urgent need for novel therapeutic strategies. Recent evidence suggests that targeting cancer cell metabolism and stress response pathways may provide promising approaches to overcome chemoresistance in ovarian cancer.

Bavachin, a bioactive phytoestrogen isolated from *Psoralea corylifolia*, has emerged as a potential therapeutic agent due to its diverse pharmacological properties. This compound has demonstrated anti-inflammatory, neuroprotective, and anti-diabetic effects through various molecular mechanisms (Takeda et al. 2018). Of particular interest, bavachin exhibits structural similarity to estrogen while showing minimal adverse effects, suggesting its potential as a selective estrogen receptor modulator (Shelly et al. 2008). Recent studies have revealed bavachin's anti-cancer activities in melanoma and hepatocellular carcinoma through modulation of mitochondrial function and endoplasmic reticulum (ER) homeostasis (Wang et al. 2016; Yang et al. 2018).

The therapeutic potential of estrogen hormone replacement therapy (HRT) in epithelial ovarian cancer has been demonstrated, with improved survival rates observed in some patients (Eeles et al. 2015). However, conventional HRT poses significant risks for various gynecological conditions (Lee, Wu, et al. 2020). Phytoestrogens may offer a safer alternative, potentially acting as estrogen antagonists while reducing the risk of endocrine-related gynecological tumors. Moreover, these compounds have shown promise as adjuvant therapies in combination with conventional chemotherapy (Virk-Baker et al. 2010).

In this study, we investigated the therapeutic potential of bavachin in ovarian cancer, focusing on three key aspects: (1) its effects on cell viability, apoptosis, and cancer stem cell properties; (2) its impact on mitochondrial function, calcium homeostasis, ER stress, and inhibition of pro-survival MAPK signaling pathways such as ERK1/2 and p38 MAPK; and (3) its potential synergistic interaction with paclitaxel. Our findings demonstrate that bavachin effectively suppresses ovarian cancer growth through multiple mechanisms and enhances the efficacy of conventional chemotherapy, suggesting its potential as a novel therapeutic strategy for ovarian cancer treatment.

## Materials and methods

### Chemicals and antibodies

Bavachin was purchased from Sigma-Aldrich (St. Louis, MO, USA). Hoechst 33342 (Cat. No: B2261) and propidium iodide (PI, Cat. No: P4170) were purchased from Sigma-Aldrich. The pan-caspase inhibitor Z-VAD-FMK (Cat. No: 627610) was obtained from Calbiochem (San Diego, CA, USA). Primary antibodies against Mcl-1, Bcl-2, Bcl-xL, PARP, cleaved PARP, GRP78/BiP, ATF4, CHOP, p-ERK1/2, ERK1/2, p-p38, p38 and β-actin were purchased from Cell Signaling Technology (Danvers, MA, USA). PD98058 (Cat. No: S1177) and SB203580 (Cat. No: S1076) were purchased from Selleck Chemicals (Houston, TX, USA).

### Cell maintenance and chemical treatment

Human ovarian cancer ES2 and OV90 cells were obtained from the American Type Culture Collection (ATCC, Manassas, VA, USA). Cells were maintained in RPMI-1640 medium supplemented with 10% fetal bovine serum (FBS; Hyclone, GE Healthcare, Chicago, IL, USA) and 1% penicillin–streptomycin in a humidified atmosphere of 5% CO_2_ at 37°C. For experiments, cells were seeded at a density of 2 × 10^4^ cells/cm^2^ and allowed to adhere overnight. Cells were then treated with various concentrations of bavachin or vehicle control for 24 h under standard culture conditions.

### Cell viability and proliferation assays

Cell viability and proliferation were assessed using two complementary methods. For trypan blue exclusion assay, ES2 and OV90 cells were harvested after bavachin treatment, stained with 0.4% trypan blue solution, and counted using a hemocytometer to determine the number of viable and dead cells. Cell viability was also evaluated by measuring cellular ATP levels using the CellTiter-Glo® Luminescent Cell Viability Assay (Promega, Madison, WI, USA) according to the manufacturer's instructions. Briefly, cells were incubated with CellTiter-Glo® reagent for 10 min at room temperature, and luminescence was measured using a microplate reader. All experiments were performed in triplicate as previously described (Lee et al. 2018). MTT assay was performed to assess cell viability. After treatment, 20 μL of MTT solution (5 mg/mL in PBS) was added to each well of a 96-well plate and incubated for 4 h at 37°C. The medium was then removed, and formazan crystals were solubilized in 100 μL of DMSO and absorbance was measured at 570 nm.

### Spheroid formation (colonosphere) assay

For three-dimensional (3D) spheroid culture, ES2 and OV90 cells were seeded at a density of 1 × 10^4^ cells per well (100 μL of 1 × 10^5^ cells/mL) in ultra-low attachment 96-well round-bottom plates. After overnight attachment, cells were treated with either vehicle or 15 μM bavachin. Spheroids were allowed to form and grow for 5 days in a humidified atmosphere of 5% CO_2_ at 37°C. Spheroid formation and growth were monitored daily using an inverted microscope.

### Analysis of apoptotic and caspase activities

Apoptotic and necrotic cell death were assessed using multiple complementary methods. For nuclear morphology analysis, cells were stained with Hoechst 33342 (10 μg/mL) and propidium iodide (PI, 5 μg/mL) for 15 min at 37°C, and visualized using a fluorescence microscope (Carl Zeiss, Oberkochen, Germany). To measure caspase-3/7 activity, the Caspase-Glo® 3/7 Assay (Promega) was performed according to the manufacturer's instructions. For caspase inhibition studies, cells were pre-treated with the pan-caspase inhibitor Z-VAD-FMK (50 μM) for 1 h prior to bavachin treatment. Apoptotic cell populations were quantified using the FITC Annexin V Apoptosis Detection Kit I (BD Biosciences, San Jose, CA, USA). Briefly, cells were harvested, washed with PBS, and stained with FITC-conjugated Annexin V and PI for 15 min at room temperature in the dark. Stained cells were analyzed using a flow cytometer. A minimum of 10,000 events were collected per sample.

### Western blot analysis

Total proteins were extracted from bavachin-treated cells using RIPA lysis buffer supplemented with protease and phosphatase inhibitors. Protein concentrations were determined using the Bradford protein assay kit (Bio-Rad, Hercules, CA, USA). Equal amounts of protein (10 μg) were separated by 10% SDS-PAGE and transferred onto nitrocellulose membranes. The membranes were blocked with 5% non-fat dry milk in TBST for 1 h at room temperature, followed by overnight incubation at 4°C with primary antibodies. After washing with TBST, membranes were incubated with HRP-conjugated secondary antibodies for 1 h at room temperature. Protein bands were visualized using Super Signal™ West Pico PLUS Chemiluminescent Substrate (Pierce, Rockford, IL, USA) and quantified using a ChemiDoc™ EQ system with Quantity One® software (Bio-Rad) as previously described (Lee et al. 2019).

### Detection of mitochondrial membrane potential and relative mitochondrial calcium levels

Mitochondrial membrane potential was assessed using the Mitochondrial Staining Kit (Sigma-Aldrich) according to the manufacturer's instructions. For mitochondrial calcium measurements, cells were loaded with Rhod-2 AM (Thermo Fisher Scientific, Waltham, MA, USA) at a final concentration of 0.1 μM for 30 min at 37°C. ES2 and OV90 cells were seeded in 6-well plates at a density of 4 × 10^4^ cells per well (2 mL of 2 × 10^4^ cells/mL) and treated with bavachin (0–15 μM) for 24 h. After staining, fluorescence intensity was analyzed using a flow cytometer as previously described (Lee et al. 2019). A minimum of 10,000 events were collected for each sample.

### Measurement of mitochondrial respiratory function

Mitochondrial respiratory function was analyzed by measuring the oxygen consumption rate (OCR) using the Seahorse XF Cell Mito Stress Test Kit (Agilent Technologies, Santa Clara, CA, USA). ES2 and OV90 cells were seeded in XF96 cell culture microplates at a density of 10,000 cells/well and treated with bavachin for 24 h. On the day of the assay, culture medium was replaced with XF DMEM medium (pH 7.4) supplemented with 10 mM glucose, 1 mM sodium pyruvate, and 2 mM L-glutamine. Cells were then sequentially exposed to oligomycin (1 μM), FCCP (1 μM), and a mixture of rotenone and antimycin A (0.5 μM each). Various parameters of mitochondrial function including basal respiration, ATP production, proton leak, maximal respiration, spare respiratory capacity, and non-mitochondrial respiration were calculated from the OCR measurements as previously described (Lee et al. 2019).

### Statistical analysis

All experiments were performed at least three times independently, and data are presented as mean ± standard deviation (SD). Statistical analyses were performed using GraphPad Prism 7 software (GraphPad Software Inc., La Jolla, CA, USA). Differences between groups were evaluated by one-way or two-way analysis of variance (ANOVA) followed by appropriate post-hoc tests as specified in the figure legends. A *P*-value < 0.05 was considered statistically significant. The exact statistical methods and significance levels are indicated in each figure legend.

## Results

### Bavachin inhibits cell viability and spheroid formation in ovarian cancer cells

**Figure 1.**
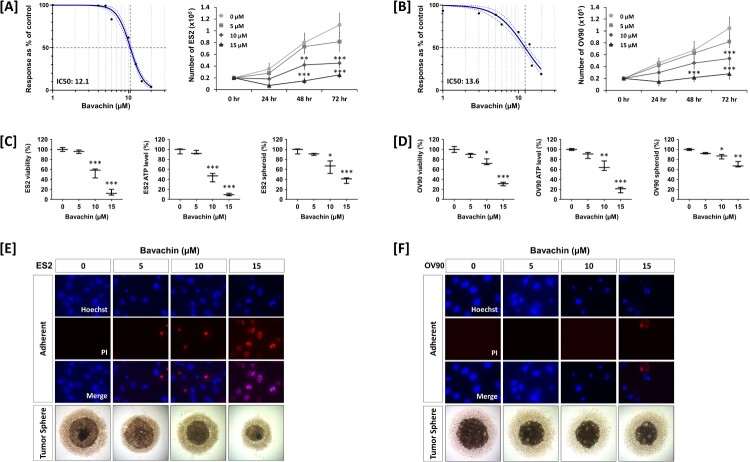
Bavachin inhibits cell viability and spheroid formation in ovarian cancer cells. (A, B) Cell viability was assessed by trypan blue exclusion assay in ES2 (A) and OV90 (B) cells treated with bavachin (0–15 μM) for 24, 48, and 72 h. IC_50_ was calculated by 24 h of bavachin treated ES2 and OV90 cells. Statistical significance was calculated in comparison to the vehicle-treated (0 μM) group at each time point. (C, D) ES2 (C) and OV90 (D) cells were treated with bavachin (0–15 μM) for 24 h. Cell viability was measured by MTT assay (left), cellular ATP levels were quantified using CellTiter-Glo® assay (middle), and spheroid formation capacity was assessed after 5 days (right). (E, F) Representative fluorescence images of Hoechst 33342/PI double staining in ES2 (E) and OV90 (F) cells treated with bavachin (0–15 μM, 24 h) under adherent (upper) or suspension (lower) conditions. Scale bars = 50 μm. Data represent mean ± SD of three independent experiments. *P < 0.05, **P < 0.01, ***P < 0.001 vs. vehicle control (one-way or two-way ANOVA followed by Sidak's multiple comparison test).

To evaluate the anti-cancer effects of bavachin, we first assessed its cytotoxicity in ES2 and OV90 ovarian cancer cells. Cells were treated with various concentrations of bavachin (1–20 μM) for 24, 48, and 72 h. Trypan blue exclusion assay revealed that bavachin inhibited cell proliferation in a time- and dose-dependent manner, with IC_50_ values of 12.1 and 13.6 μM for ES2 and OV90 cells, respectively (Figure 1(A,B)). For IC_50_ determination shown in Figure 1(A,B), cells were treated with bavachin for 24 h, and the dose response curve was generated using various concentrations ranging from 0 to 15 μM. To further confirm these effects, we measured cell viability using two complementary methods. The MTT assay showed that bavachin treatment significantly reduced the viability of both ES2 and OV90 cells (Figure 1(C)). Consistently, cellular ATP levels were markedly decreased in both cell lines after treatment with 10 μM bavachin (Figure 1(D)).

Given that cancer stem cells play crucial roles in tumor progression and drug resistance, we next investigated the effect of bavachin on spheroid formation capacity. Three-dimensional spheroid cultures of ES2 and OV90 cells were treated with 15 μM bavachin for 5 days. Bavachin treatment significantly suppressed spheroid formation in both cell lines (Figure 1(E,F)), suggesting its potential efficacy against cancer stem-like populations. These results collectively demonstrate that bavachin effectively inhibits both proliferation and stemness properties of ovarian cancer cells.

### Bavachin induces caspase-dependent apoptosis in ovarian cancer cells

**Figure 2.**
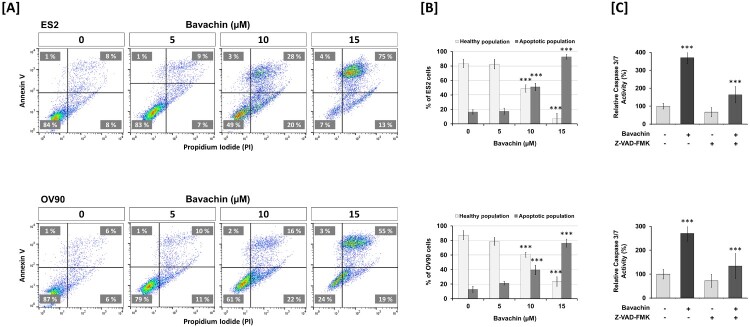
Bavachin induces caspase-dependent apoptosis in ovarian cancer cells. (A) Flow cytometric analysis of cell death using Annexin V-FITC/PI staining in ES2 (upper) and OV90 (lower) cells treated with bavachin (0–15 μM, 24 h). (B) Quantification of live (Annexin V-/PI-) and apoptotic (Annexin V+) cell populations. Statistical comparisons were performed relative to the untreated control (0 μM bavachin). (C) Caspase-3/7 activity in cells treated with bavachin (15 μM) ± Z-VAD-FMK (50 μM, 1 h pretreatment). Statistical comparisons were performed relative to the bavachin-only treatment. Data represent mean ± SD of three independent experiments. *P < 0.05, **P < 0.01, ***P < 0.001 (one-way or two-way ANOVA followed by Sidak's multiple comparison test).

To elucidate the mechanism underlying bavachin-induced cell death, we first examined nuclear morphological changes using Hoechst 33342 and PI double staining. Bavachin-treated ES2 and OV90 cells exhibited characteristic apoptotic features including nuclear fragmentation and cellular shrinkage (Figure 1(E, F)). To quantify the apoptotic cell population, we performed flow cytometric analysis using Annexin V/PI staining. Bavachin treatment increased the percentage of apoptotic cells in a dose-dependent manner, with a concurrent decrease in viable cell populations in both ES2 and OV90 cells (Figure 2(A, B)).

To determine whether bavachin-induced apoptosis was caspase-dependent, we measured caspase-3/7 activity and examined the effect of the pan-caspase inhibitor Z-VAD-FMK. Bavachin treatment significantly increased caspase-3/7 activity by 3.7- and 2.9-fold in ES2 and OV90 cells, respectively. Notably, pretreatment with Z-VAD-FMK substantially suppressed bavachin-induced caspase activation in both cell lines (Figure 2(C)).

**Figure 3.**
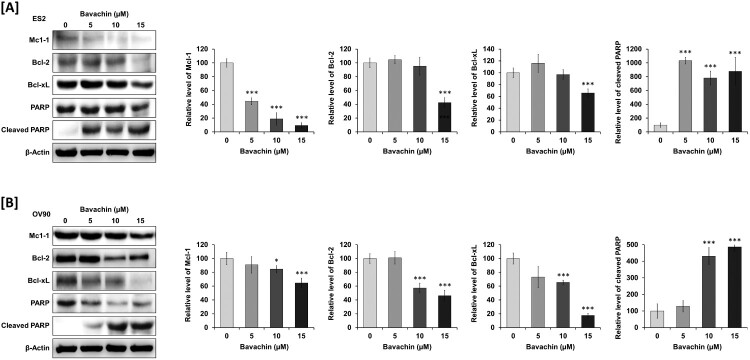
Bavachin modulates apoptosis-related protein expression in ovarian cancer cells. (A, B) Western blot analysis of anti-apoptotic proteins (Mcl-1, Bcl-2, Bcl-xL) and PARP cleavage in ES2 (A) and OV90 (B) cells treated with bavachin (0–15 μM, 24 h). Left panels show representative immunoblots; right panels show densitometric analysis normalized to β-actin. Data represent mean ± SD of three independent experiments. *P < 0.05, **P < 0.01, ***P < 0.001 vs. vehicle control (one-way ANOVA followed by Sidak's multiple comparison test).

We further investigated the molecular mechanisms by analyzing the expression of apoptosis-related proteins. Western blot analysis revealed that bavachin treatment significantly downregulated anti-apoptotic proteins including Mcl-1 (10.7- and 1.5-fold), Bcl-2 (2.4- and 2.2-fold), and Bcl-xL (1.5- and 5.7-fold) in ES2 and OV90 cells, respectively (Figure 3(A, B)). Concurrently, bavachin markedly increased the levels of cleaved PARP by 880- and 490-fold in ES2 and OV90 cells, respectively. These results collectively demonstrate that bavachin induces apoptosis through activation of the caspase-dependent pathway and modulation of apoptosis-related proteins in ovarian cancer cells.

### Bavachin disrupts mitochondrial function through membrane depolarization and calcium dysregulation

**Figure 4.**
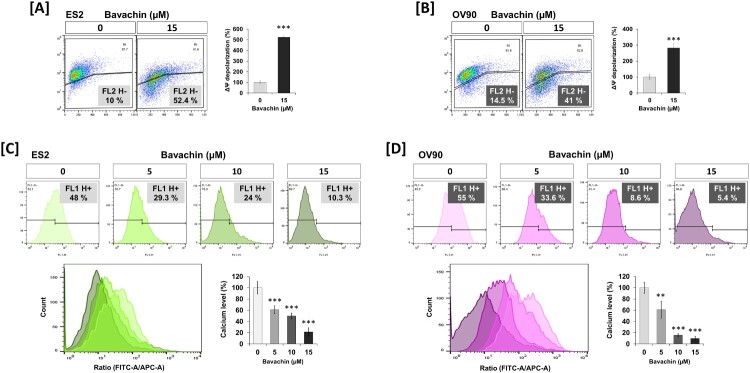
Bavachin disrupts mitochondrial membrane potential and calcium homeostasis. (A, B) Flow cytometric analysis of mitochondrial membrane potential (ΔΨm) using JC-1 staining in ES2 (A) and OV90 (B) cells treated with bavachin (15 μM, 24 h). Right panels show quantification of cells with depolarized mitochondria indicated by FL2-H. Left panels graph quantified the relative values of calcium levels based on 0 μM as 100%. (C, D) Mitochondrial calcium levels assessed by Rhod-2 AM staining in ES2 (C) and OV90 (D) cells treated with bavachin (15 μM, 24 h). Flow cytometry histograms (left) and quantification of fluorescence intensity (right) are shown. Data represent mean ± SD of three independent experiments. **P < 0.01, ***P < 0.001 vs. vehicle control (Student's t-test).

To investigate the effects of bavachin on mitochondrial function, we first assessed changes in mitochondrial membrane potential (Δψm) using a cationic fluorescent dye. Treatment with bavachin (15 μM, 24 h) induced significant mitochondrial membrane depolarization in both ES2 and OV90 cells, as evidenced by a decrease in fluorescence intensity, respectively (*P* < 0.01; Figure 4(A, B)). This reduction in Δψm, together with the previously observed decrease in cellular ATP levels (Figure 1(C, D)), suggested substantial impairment of mitochondrial function.

Given that mitochondrial calcium homeostasis is closely linked to membrane potential and cellular energy status, we next examined mitochondrial calcium levels using Rhod-2 AM fluorescence. Flow cytometric analysis revealed that bavachin treatment significantly increased mitochondrial calcium accumulation in ES2 and OV90 cells, respectively (*P* < 0.01; Figure 4(D, E)). This calcium overload, occurring concurrently with membrane depolarization, suggests that bavachin disrupts mitochondrial calcium homeostasis, which may contribute to the loss of mitochondrial integrity and subsequent activation of the apoptotic pathway.

### Bavachin alters mitochondrial metabolism and energy phenotype in ovarian cancer cells

**Figure 5.**
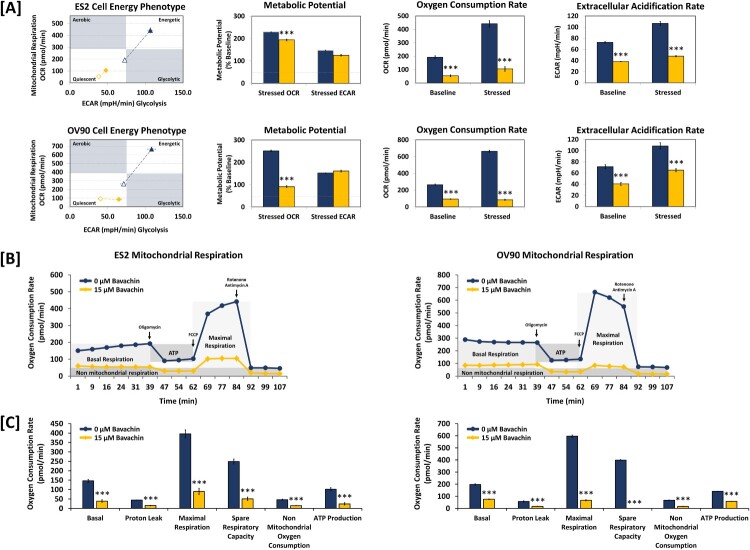
Bavachin impairs mitochondrial respiration and cellular bioenergetics. (A) Energy phenotype analysis of ES2 (upper) and OV90 (lower) cells treated with bavachin (15 μM, 24 h) using Seahorse XF analyzer. (B) Real-time oxygen consumption rate (OCR) measurements during sequential injection of oligomycin (1 μM), FCCP (1 μM), and rotenone/antimycin A (0.5 μM each) in ES2 (left) and OV90 (right) cells. (C) Quantification of specific OCR parameters in ES2 (left) and OV90 (right) cells. In bar graphs shown in (A) and (C), black bars represent the vehicle control (0 μM bavachin), and gray bars represent bavachin-treated cells (15 μM). Data represent mean ± SD of three independent experiments. *P < 0.05, **P < 0.01, ***P < 0.001 vs. vehicle control (two-way ANOVA followed by Dunnett's test).

Given the observed effects of bavachin on mitochondrial membrane potential and ATP production, we next investigated its impact on cellular energy metabolism. Using the Seahorse XF analyzer, we first examined the energy phenotype of ES2 and OV90 cells following bavachin treatment (15 μM, 24 h). Bavachin significantly reduced both oxygen consumption rate (OCR) and extracellular acidification rate (ECAR), indicating a concurrent suppression of oxidative phosphorylation and glycolysis. This metabolic suppression shifted the cellular energy phenotype from an energetic to a quiescent state (Figure 5(A)).

To further characterize the effects on mitochondrial function, we performed a mitochondrial stress test by sequential addition of specific modulators of the electron transport chain (ETC). While vehicle-treated cells showed characteristic OCR responses to oligomycin, FCCP, and rotenone/antimycin A, bavachin-treated cells exhibited markedly diminished responses to these compounds in both cell lines (Figure 5(B)). Detailed analysis of specific OCR parameters revealed significant reductions in basal respiration, ATP production, maximal respiration, spare respiratory capacity, proton leak, and non-mitochondrial oxygen consumption in bavachin-treated cells compared to controls (*P* < 0.001; Figure 5(C)). These results demonstrate that bavachin comprehensively impairs cellular bioenergetics by simultaneously disrupting both oxidative phosphorylation and glycolytic pathways, ultimately leading to metabolic catastrophe in ovarian cancer cells.

### Bavachin activates the ER stress pathway in ovarian cancer cells

**Figure 6.**
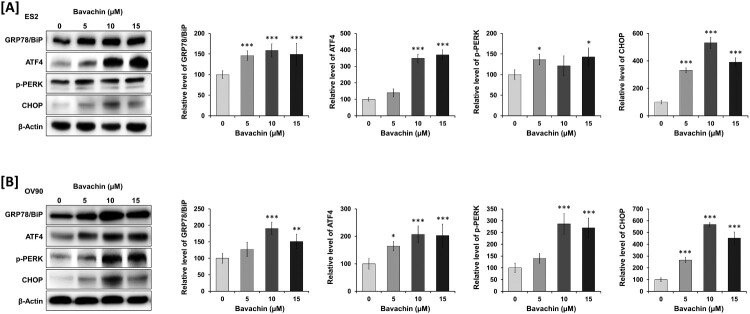
Bavachin activates the ER stress pathway. (A, B) Western blot analysis of ER stress markers (GRP78, ATF4, PERK, and CHOP) in ES2 (A) and OV90 (B) cells treated with bavachin (0–15 μM, 24 h). Left panels show representative immunoblots; right panels show densitometric analysis normalized to β-actin. Data represent mean ± SD of three independent experiments. *P < 0.05, **P < 0.01, ***P < 0.001 vs. vehicle control (one-way ANOVA followed by Sidak's multiple comparison test).

Given the close functional relationship between mitochondria and the endoplasmic reticulum (ER), we next investigated whether bavachin affects ER stress signaling. Western blot analysis revealed that bavachin treatment (0–15 μM, 24 h) induced a dose-dependent increase in ER stress-related proteins in both ES2 and OV90 cells. In ES2 cells, bavachin significantly upregulated the expression of the ER stress sensor GRP78 (1.6-fold), followed by increased levels of downstream effectors including ATF4 (3.6-fold), PERK (1.4-fold), and CHOP (3.9-fold) compared to vehicle control (*P* < 0.01; Figure 6(A)). Similarly, OV90 cells showed elevated expression of GRP78 (1.6-fold), ATF4 (2.1-fold), PERK (2.7-fold), and CHOP (4.5-fold) following bavachin treatment (*P* < 0.01; Figure 6(B)). These results demonstrate that bavachin activates the canonical ER stress pathway, as evidenced by the sequential upregulation of GRP78 and its downstream targets, suggesting that ER stress may contribute to bavachin-induced cell death in ovarian cancer cells.

### Bavachin regulates ERK1/2 and p38 MAPK signaling pathways in ovarian cancer cells

**Figure 7.**
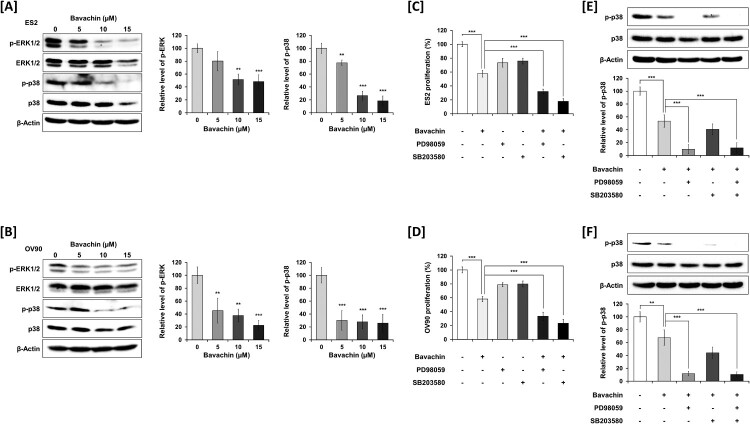
Bavachin suppresses ERK1/2 and p38 MAPK signaling in ovarian cancer cells. (A, B) Western blot analysis of phosphorylated- and total-ERK1/2 and p38 MAPK in ES2 and OV90 cells treated with bavachin for 24 h in ES2 (A) and OV90 cells. Left panels show representative immunoblots; right panels show densitometric analysis normalized to β-actin. (C, D) Effects of treatment with bavachin and ERK inhibitors (PD98059, 10 µM) or p38 MAPK inhibitors (SB203580, 20 µM) on cell proliferation in ES2 (C) and OV90 (D) ovarian cancer cells. (E, F) Western blot analysis of phosphorylation- and total-p38 MAPK in treated cells for 24 h with bavachin and ERK inhibitors (PD98059, 10 µM) or p38 MAPK inhibitors (SB203580, 20 µM) in ES2 (E) and OV90 (F) cells. The upper panel shows a representative immunoblot, and the lower panel shows a densitometric analysis normalized with β-actin. **P < 0.01, ***P < 0.001 vs. vehicle control (one-way ANOVA followed by Sidak's multiple comparison test).

To further elucidate the mechanisms underlying bavachin-induced cytotoxicity, we investigated its effects on mitogen-activated protein kinase (MAPK) signaling pathways. Western blot analysis demonstrated that bavachin treatment (0–15 μM for 24 h) markedly reduced the phosphorylation levels of ERK1/2 and p38 MAPK in both ES2 (Figure 7(A)) and OV90 (Figure 7(B)) ovarian cancer cells, without affecting the expression of total ERK1/2 and p38 MAPK. Densitometric quantification confirmed that treatment with 15 μM bavachin resulted in a > 50% reduction in phospho-ERK1/2 and phospho-p38 MAPK levels compared to control. To further comprehensive demonstration for these pathways in bavachin-mediated cancer proliferation, cell growth was analyzed using specific inhibitors of ERK1/2 (PD98059, 10 μM) and p38 MAPK (SB203580, 20 μM). Co-treatment with bavachin and either PD98059 or SB203580 led to a more pronounced suppression of proliferation in both ES2 (Figure 7(C)) and OV90 (Figure 7(D)) cells compared to bavachin treatment alone. Consistent with these findings, Western blot analysis revealed that co-treatment with bavachin and either inhibitor resulted in further suppression of phospho-p38 MAPK levels compared to bavachin alone in ES2 (Figure 7(E)) and OV90 (Figure 7(F)) cells. Collectively, these results suggest that bavachin inhibits MAPK-mediated prosurvival signaling cascades, thereby contributing to its pro-apoptotic and chemosensitizing effects in ovarian cancer cells.

### Bavachin enhances paclitaxel sensitivity in ovarian cancer cells

**Figure 8.**
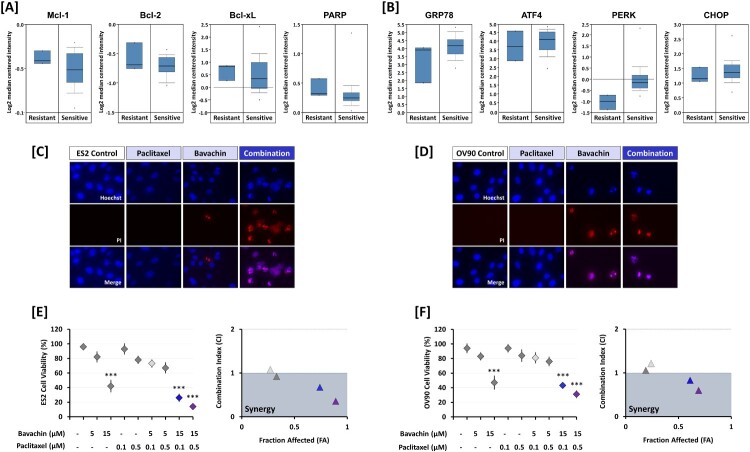
Bavachin enhances paclitaxel sensitivity in ovarian cancer cells. (A, B) ONCOMINE analysis comparing expression levels of apoptosis-related genes (A) and ER stress-related genes (B) between paclitaxel-sensitive and -resistant ovarian cancer patients. (C, D) Representative fluorescence images of Hoechst 33342/PI staining in ES2 (C) and OV90 (D) cells treated with bavachin (15 μM) ± paclitaxel (0.5 μM) for 24 h. Scale bars = 50 μm. (E, F) Combination index (CI) analysis in ES2 (E) and OV90 (F) cells treated with various combinations of bavachin (5, 15 μM) and paclitaxel (0.1, 0.5 μM). CI values were calculated using CompuSyn software. The statistical comparisons for combination treatments (bavachin + paclitaxel) were performed against each single treatment group, respectively. For bavachin (15 μM) alone, the comparison was made against the untreated control group. Data represent mean ± SD of three independent experiments. (two-way ANOVA followed by Sidak's multiple comparison test).

To investigate the potential therapeutic applications of bavachin, we examined whether it could enhance the efficacy of paclitaxel, a standard chemotherapeutic agent for ovarian cancer. Analysis of ONCOMINE dataset revealed that paclitaxel-resistant ovarian cancer patients showed significantly higher expression of anti-apoptotic genes (*Mcl-1*, *Bcl-2*, *Bcl-xL*, and *PARP*) compared to paclitaxel-sensitive patients (*P* < 0.01; Figure 8(A)). Conversely, ER stress-related genes were significantly upregulated in paclitaxel-sensitive patients (*P* < 0.01; Figure 7(B)), suggesting that modulation of these pathways might influence paclitaxel sensitivity. Based on these observations and our findings that bavachin downregulates anti-apoptotic proteins while inducing ER stress, we evaluated the combination effects of bavachin and paclitaxel. Co-treatment with bavachin (15 μM) and paclitaxel (0.1 μM) significantly enhanced cell death compared to either agent alone, as assessed by Hoechst 33342/PI staining and cell viability assays (*P* < 0.001; Figure 8(C, D)). To quantify this interaction, we calculated combination index (CI) values using the Chou-Talalay method. The CI values were less than 1 (ES2: 0.23; OV90: 0.66) in both cell lines, indicating a synergistic interaction between bavachin and paclitaxel (Figure 8(E, F)). These results suggest that bavachin may enhance the therapeutic efficacy of paclitaxel, potentially through concurrent modulation of apoptotic and ER stress pathways.

**Figure 9.**
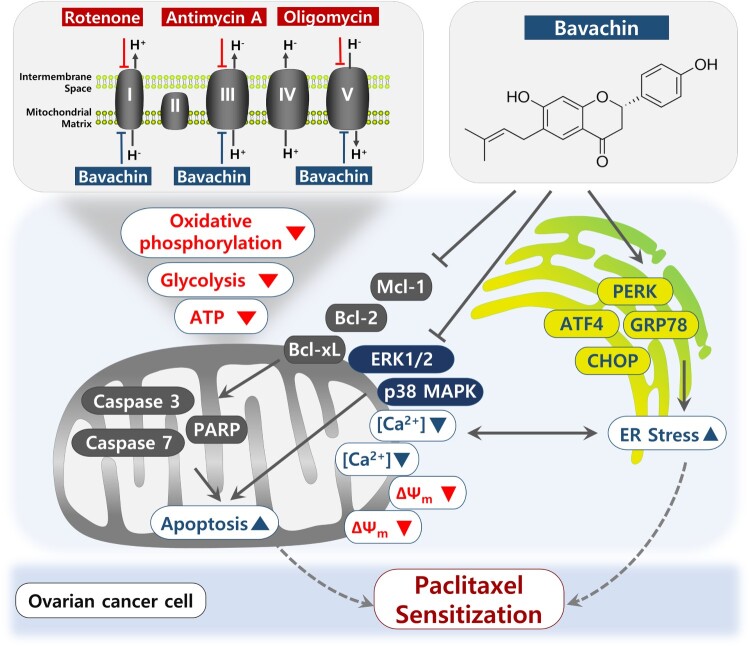
Proposed mechanism of bavachin-mediated anti-cancer effects in ovarian cancer. Schematic illustration showing bavachin-induced apoptosis through modulation of mitochondrial function, ER stress, and oncogenic ERK1/2 and p38 MAPK signaling, enhancing apoptosis and cellular metabolism in ovarian cancer cells. The combination of bavachin with paclitaxel leads to enhanced therapeutic efficacy through synergistic activation of cell death pathways.

A schematic summary of the proposed mechanism is illustrated in Figure 9. Bavachin induces apoptosis in ovarian cancer cells through disruption of mitochondrial function, induction of ER stress, and inhibition of oncogenic ERK1/2 and p38 MAPK signaling pathways. Furthermore, its combination with paclitaxel results in synergistic activation of cell apoptotic pathways, ultimately enhancing therapeutic efficacy.

## Discussion

Ovarian cancer remains a significant therapeutic challenge, with platinum- and taxane-based regimens serving as first-line treatments despite their limitations of drug resistance and toxicity (Virk-Baker et al. 2010; Loret et al. 2019). Here, we demonstrate that bavachin, a natural phytoestrogen, exhibits potent anti-cancer activity in ovarian cancer cells through multiple mechanisms and potentially enhances paclitaxel sensitivity.

Our study reveals that bavachin's anti-cancer effects are primarily mediated through disruption of cellular bioenergetics and stress responses. Specifically, bavachin induced mitochondrial dysfunction, characterized by membrane depolarization and calcium dysregulation, leading to comprehensive impairment of both oxidative phosphorylation and glycolysis. This finding aligns with emerging evidence challenging the Warburg effect paradigm, suggesting that targeting mitochondrial function represents a promising therapeutic strategy in cancer (Nunnari and Suomalainen 2012). Similar to metformin's mechanism of action in cancer cells through inhibition of mitochondrial complex I (Wheaton et al. 2014), bavachin's ability to disrupt mitochondrial function may contribute to its therapeutic efficacy.

The role of phytoestrogens in cancer treatment has gained increasing attention as alternatives to conventional hormone therapy, which can increase cancer risk (Pritchard 2001). Previous studies have demonstrated diverse mechanisms of action for phytoestrogens, including modulation of hydroxysteroid dehydrogenases, antioxidant properties, and inhibition of various signaling pathways (Krazeisen et al. 2001; Barnes 2010). Bavachin, in particular, has shown therapeutic potential in multiple diseases through various mechanisms, including mTOR-related autophagy and β-catenin pathway regulation (He et al. 2019). Our findings extend bavachin's therapeutic profile to ovarian cancer, demonstrating its ability to induce ER stress and apoptosis while suppressing cancer stem cell-like properties.

In a previous study conducted in human placental choriocarcinoma cells, Bavachin was shown to induce caspase-dependent apoptosis, mitochondrial respiratory suppression, endoplasmic reticulum (ER) stress, and disruption of calcium homeostasis (Lee, Lim, et al. 2020). While these mechanisms contribute to the general understanding of Bavachin's anti-cancer potential, they do not fully explain its effects in different tumor types. In this study, we have newly identified that bavachin suppresses phosphorylation of ERK1/2 and p38MAPK in ovarian cancer cells. Both ERK1/2 and p38MAPK are critical components of the MAPK signaling cascade that regulate cell survival, proliferation, and stress response. Inhibition of these pro-survival pathways suggests that bavachin exerts its cytotoxic effects not only by triggering apoptosis via intrinsic and ER-stress pathways, but also by directly targeting oncogenic signaling pathways including ERK and MAPK. These findings align with the emerging view that targeting MAPK signaling may overcome chemoresistance and enhance therapeutic efficacy in ovarian cancer. This novel insight into the regulation of the ERK and p38 MAPK pathways by Bavachin underscores its therapeutic potential in the treatment of ovarian cancer and highlights a mechanistic distinction from previously reported effects in other cell types. The addition of this novel mechanism significantly strengthens the therapeutic relevance of bavachin.

Notably, our analysis of clinical data revealed that paclitaxel resistance in ovarian cancer patients correlates with increased anti-apoptotic gene expression and suppressed ER stress pathways. Given that bavachin effectively modulates both these pathways, we investigated its potential to enhance paclitaxel sensitivity. Indeed, the combination of bavachin and paclitaxel showed synergistic anti-cancer effects, suggesting a promising strategy to overcome drug resistance. This is particularly significant considering the limited clinical response rates to paclitaxel monotherapy (Trope et al. 1997) and the toxicity concerns associated with current combination approaches (Bolis et al. 2010).

In conclusion, our study demonstrates that bavachin effectively suppresses ovarian cancer cell growth through multiple mechanisms, including disruption of mitochondrial bioenergetics, activation of ER stress, and inhibition of prosurvival MAPK signaling pathways such as ERK1/2 and p38 MAPK. These molecular alterations collectively contribute to the activation of apoptotic pathways and inhibition of tumor cell proliferation. Importantly, bavachin not only exhibits potent anti-cancer activity as a single agent, but also synergistically enhances the cytotoxicity of paclitaxel, a standard chemotherapeutic agent used in ovarian cancer treatment.

A schematic illustration of the proposed mechanism is presented in Figure 9, summarizing how bavachin mediates apoptosis through integrated modulation of mitochondrial function, ER stress, and MAPK signaling, ultimately amplifying cell death responses. The combination of bavachin with paclitaxel further potentiates these effects, underscoring its potential as an adjuvant therapy to improve the efficacy of conventional chemotherapy.

These findings provide a strong rationale for further investigation of bavachin as a novel therapeutic strategy, particularly in the context of combination treatments aimed at overcoming chemoresistance and enhancing therapeutic outcomes in ovarian cancer patients.

## Author contributions

JYL and KKK conceived and designed the culture experiments, the cell culture methodology, and all other experiments; SJL collected experimental samples and conducted all experiments; SJL and JYL analyzed and interpreted the data and contributed to the development of the manuscript. All authors contributed to its critical review and agreed on the final version.

## References

[CIT0001] Barnes S. 2010. The biochemistry, chemistry and physiology of the isoflavones in soybeans and their food products. Lymphat Res Biol. 8:89–98. doi:10.1089/lrb.2009.0030. Epub 2010/03/20. 20235891 PMC2883528

[CIT0002] Bolis G, Scarfone G, Raspagliesi F, Mangili G, Danese S, Scollo P, Lo Russo D, Villa A, Aimone PD, Scambia G. 2010. Paclitaxel/carboplatin versus topotecan/paclitaxel/carboplatin in patients with FIGO suboptimally resected stage III-IV epithelial ovarian cancer a multicenter, randomized study. Eur J Cancer. 46:2905–2912. doi:10.1016/j.ejca.2010.06.124. Epub 2010/08/03. 20673626

[CIT0003] Eeles RA, Morden JP, Gore M, Mansi J, Glees J, Wenczl M, Williams C, Kitchener H, Osborne R, Guthrie D, et al. 2015. Adjuvant hormone therapy may improve survival in epithelial ovarian cancer: results of the AHT randomized trial. J Clin Oncol. 33:4138–4144. doi:10.1200/JCO.2015.60.9719. Epub 2015/09/30. 26417001

[CIT0004] He HQ, Law BYK, Zhang N, Qiu CL, Qu YQ, Wu AG, Han Y, Song Q, Zheng WL, Liu Y, et al. 2019. Bavachin protects human aortic smooth muscle cells against beta-glycerophosphate-mediated vascular calcification and apoptosis via activation of mTOR-dependent autophagy and suppression of beta-catenin signaling. Front Pharmacol. 10:1427. doi:10.3389/fphar.2019.01427. Epub 2020/01/11. 31920640 PMC6930901

[CIT0005] Krazeisen A, Breitling R, Moller G, Adamski J. 2001. Phytoestrogens inhibit human 17beta-hydroxysteroid dehydrogenase type 5. Mol Cell Endocrinol. 171:151–162. doi:10.1016/S0303-7207(00)00422-6. Epub 2001/02/13. 11165023

[CIT0006] Lee AW, Wu AH, Wiensch A, Mukherjee B, Terry KL, Harris HR, Carney ME, Jensen A, Cramer DW, Berchuck A, et al. 2020. Estrogen plus progestin hormone therapy and ovarian cancer: a complicated relationship explored. Epidemiology. 31(3):402–408. doi:10.1097/EDE.0000000000001175. Epub 2020/02/07. 32028322 PMC7584395

[CIT0007] Lee JY, Lim W, Park S, Kim J, You S, Song G. 2019. Deoxynivalenol induces apoptosis and disrupts cellular homeostasis through MAPK signaling pathways in bovine mammary epithelial cells. Environ Pollut. 252:879–887. doi:10.1016/j.envpol.2019.06.001. Epub 2019/06/17. 31203115

[CIT0008] Lee JY, Lim W, Song G. 2020. Bavachin suppresses human placental choriocarcinoma cells by targeting electron transport chain complexes and mitochondrial dysfunction. Free Radic Biol Med. 156:26–35. doi:10.1016/j.freeradbiomed.2020.05.022. Epub 2020/06/09. 32505737

[CIT0009] Lee JY, Talhi O, Jang D, Cerella C, Gaigneaux A, Kim KW, Lee JW, Dicato M, Bachari K, Han BW, et al. 2018. Cytostatic hydroxycoumarin OT52 induces ER/Golgi stress and STAT3 inhibition triggering non-canonical cell death and synergy with BH3 mimetics in lung cancer. Cancer Lett. 416:94–108. doi:10.1016/j.canlet.2017.12.007. Epub 2017/12/17. 29247826

[CIT0010] Loret N, Denys H, Tummers P, Berx G. 2019. The role of epithelial-to-mesenchymal plasticity in ovarian cancer progression and therapy resistance. Cancers. 11(6):838. doi:10.3390/cancers11060838. Epub 2019/06/20. 31213009 PMC6628067

[CIT0011] Nunnari J, Suomalainen A. 2012. Mitochondria: in sickness and in health. Cell. 148:1145–1159. doi:10.1016/j.cell.2012.02.035. Epub 2012/03/20. 22424226 PMC5381524

[CIT0012] Pritchard KI. 2001. Breast cancer prevention with selective estrogen receptor modulators: a perspective. Ann N Y Acad Sci. 949:89–98. doi:10.1111/j.1749-6632.2001.tb04006.x. Epub 2002/01/25. 11795385

[CIT0013] Shelly W, Draper MW, Krishnan V, Wong M, Jaffe RB. 2008. Selective estrogen receptor modulators: an update on recent clinical findings. Obstet Gynecol Surv. 63:163–181. doi:10.1097/OGX.0b013e31816400d7. Epub 2008/02/19. 18279543

[CIT0014] Takeda T, Tsubaki M, Tomonari Y, Kawashima K, Itoh T, Imano M, Satou T, Nishida S. 2018. Bavachin induces the apoptosis of multiple myeloma cell lines by inhibiting the activation of nuclear factor kappa B and signal transducer and activator of transcription 3. Biomed Pharmacother. 100:486–494. doi:10.1016/j.biopha.2018.02.019. Epub 2018/02/27. 29477912

[CIT0015] Trope C, Kaern J, Kristensen G, Rosenberg P, Sorbe B. 1997. Paclitaxel in untreated FIGO stage III suboptimally resected ovarian cancer. Ann Oncol. 8:803–806. doi:10.1023/A:1008230909599. Epub 1997/08/01. 9332691

[CIT0016] Virk-Baker MK, Nagy TR, Barnes S. 2010. Role of phytoestrogens in cancer therapy. Planta Med. 76:1132–1142. doi:10.1055/s-0030-1250074. Epub 2010/07/03. 20597043 PMC3800092

[CIT0017] Wang JH, Pei YY, Xu HD, Li LJ, Wang YQ, Liu GL, Qu Y, Zhang N. 2016. Effects of bavachin and its regulation of melanin synthesis in A375 cells. Biomed Rep. 5:87–92. doi:10.3892/br.2016.688. Epub 2016/06/28. 27347410 PMC4906567

[CIT0018] Wheaton WW, Weinberg SE, Hamanaka RB, Soberanes S, Sullivan LB, Anso E, Glasauer A, Dufour E, Mutlu GM, Budigner GS, et al. 2014. Metformin inhibits mitochondrial complex I of cancer cells to reduce tumorigenesis. Elife. 3:e02242. doi:10.7554/eLife.02242s. Epub 2014/05/21. 24843020 PMC4017650

[CIT0019] Yang Y, Tang X, Hao F, Ma Z, Wang Y, Wang L, Gao Y. 2018. Bavachin induces apoptosis through mitochondrial regulated ER stress pathway in HepG2 cells. Biol Pharm Bull. 41:198–207. doi:10.1248/bpb.b17-00672. Epub 2017/12/01. 29187671

